# Infective Endocarditis Leading to Intracranial Abscess: A Case Report and Literature Review

**DOI:** 10.7759/cureus.12660

**Published:** 2021-01-12

**Authors:** Shafaq Taj, Muhammad Usman Arshad, Hira Khan, Guneet S Sidhu, Romil Singh

**Affiliations:** 1 Internal Medicine, Deccan College of Medical Sciences, Hyderabad, IND; 2 Department of Internal Medicine, Allama Iqbal Medical College, Jinnah Hospital Lahore, Lahore, PAK; 3 Internal Medicine, Islamic International Medical College, Rawalpindi, PAK; 4 Department of Gastroenterology and Hepatology, Mayo Clinic, Rochester, USA; 5 Critical Care, Mayo Clinic, Rochester, USA

**Keywords:** infective endocarditis, intracranial abscess, septic emboli, vegetation

## Abstract

Neurologic complications are a hallmark of infective endocarditis (IE). IE leading to intracranial abscess has an unfavorable prognosis. A 17-year-old boy with a past medical history of aortic valve replacement presented with fever and seizure. On examination, he had tachycardia, systolic murmur, slurred speech, meningeal signs, and right homonymous hemianopia. His laboratory analysis revealed an elevated erythrocyte sedimentation rate and C-reactive protein. The brain's magnetic resonance imaging revealed multiple ring-enhancing lesions in the frontal, occipital lobe, and occipitotemporal lobe, consistent with intracranial abscess. Transthoracic echocardiogram revealed a mobile mass adjacent to aortic value, consistent with possible infective vegetation. He was diagnosed with multiple cerebral septic emboli leading to intracranial abscess due to IE. Blood and cerebrospinal fluid culture revealed methicillin-sensitive *Staphylococcus aureus* growth. He was started on intravenous nafcillin and gentamycin. His condition improved gradually, and he became afebrile on hospital day four. On his recent follow-up, he was doing well.

## Introduction

Infective endocarditis (IE) is linked to significant cardiac and non-cardiac morbidity. Neurologic complications of IE have the worst prognosis of all. IE leading septic embolism is the only complication, which has the possibility of causing overwhelming sequel and even life-threatening circumstances. IE has a diverse presentation and can involve different body organs and body systems in patients with predisposing conditions. Septic emboli from the septic vegetation can spread to the brain, kidney, spleen, and lungs resulting in massive metastatic infections. Neurological consequences in IE are common and have worse long-term outcomes. These complications may include meningitis, brain abscess, ischemic stroke, and mycotic aneurysm. Among the neurological complications, brain abscess is rare. *Staphylococcus aureus* (*S. aureus*) is the most common causative agent of IE. Herein we present a case of IE and brain abscess as a non-cardiac complication of IE caused by the spread of the multiple septic emboli.

## Case presentation

A 17-year-old boy presented to the emergency department with fever and mental status change, associated with rigors and chills, nausea, vomiting, arthralgia, and generalized headache. He was a known case of sub-aortic stenosis in his early life and managed with surgery at four. Later on, he developed severe aortic stenosis at the age of seven, and he was treated with aortic valvuloplasty. He remained asymptomatic after that surgery until today’s presentation. He had no history of any seizure, surgery, or dental surgery and denied chest pain, palpitation, and shortness of breath. Upon initial evaluation, he had a temperature of 101^o^F, blood pressure of 100/70 mmHg, respiratory rate of 26 minutes, heart rate of 105 per minute, and oxygen saturation of 99%.

On examination, he was confused, sleepy, and well oriented in time and place but not in person. He had no skin manifestation of endocarditis. The cardiovascular examination revealed tachycardia and a systolic murmur. The respiratory and gastrointestinal review was nonsignificant. On neurological examination, he had slurred speech and meningeal signs with unremarkable motor and sensory analysis. Cranial nerves were intact except the optic nerve, which revealed right homonymous hemianopia. His initial laboratory showed an elevated erythrocyte sedimentation rate (ESR) and C-reactive protein (CRP) of 95 mm/hr and 13 mg/dL, respectively. His thyroid-stimulating hormone (TSH) level was nonsignificant, and Epstein-Barr virus (EBV), human immunodeficiency virus (HIV), and monospot tests all were negative.

**Figure 1:**
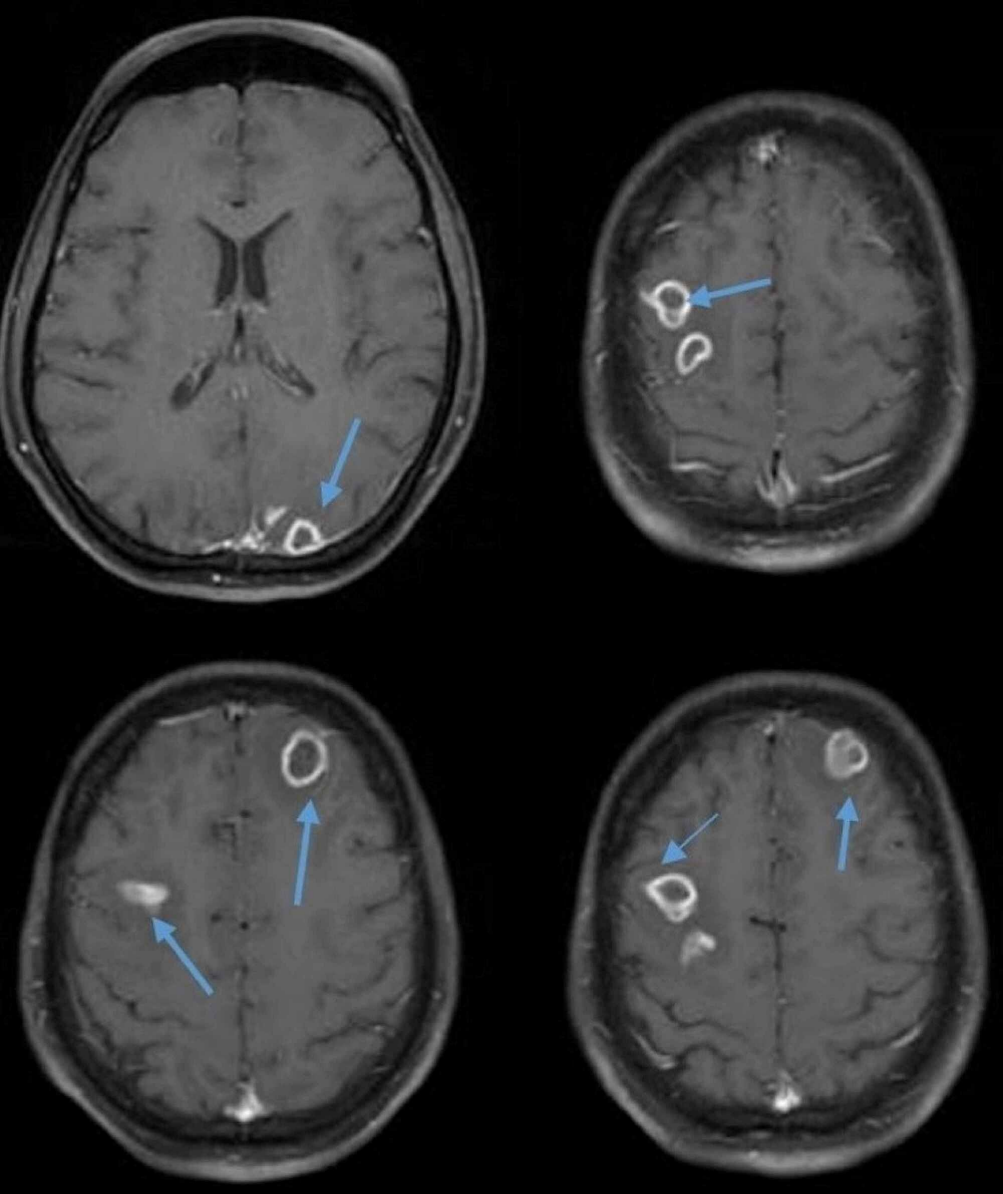
Axial images of brain MRI showing multiple ring-enhancing lesions of various sizes in the frontal, parietal, and temporal lobe (blue arrows). MRI: Magnetic resonance imaging

**Figure 2:**
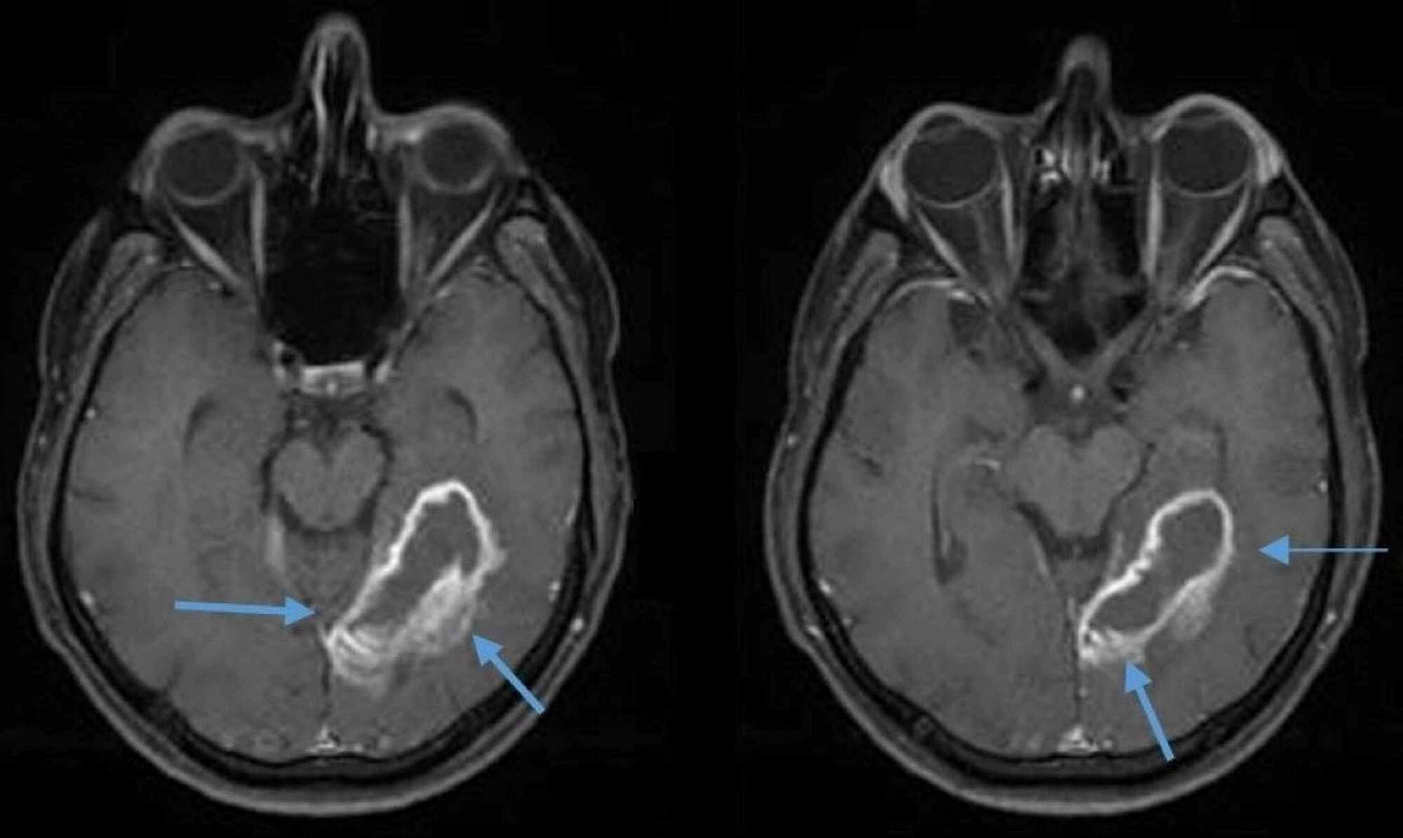
Axial images of the brain MRI showing a ring-enhancing lesion in the left occipitotemporal lobe with significant surrounding vasogenic edema (blue arrows). MRI: Magnetic resonance imaging

His clinical picture was suggestive of a central lesion. Brain magnetic resonance imaging (MRI) was performed, revealing multiple round ring-enhancing lesions in the frontal, occipital lobe, and temporooccipital lobe (Figure 1). These lesions were isointense on T1 weighted (T1W1) image, hyperintense in T2 weighted (T2W2) image, with the largest lesion measuring 4.6x1.0 cm with a wall thickness of 3 mm in the left occipitotemporal lobe with significant surrounding vasogenic edema, consistent with septic emboli (Figure 2). Additional imaging with computed tomography (CT) of the chest, abdomen, and pelvis was nonsignificant for possible infection and emboli sources.

**Figure 3:**
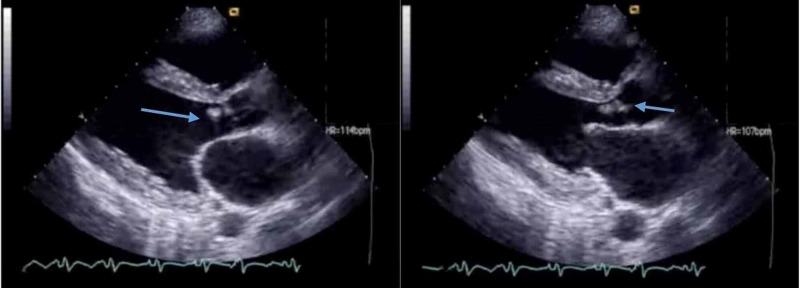
Echocardiography showing aortic valve vegetations consistent with possible infective endocarditis (blue arrows).

The patient underwent echocardiography, which revealed aortic regurgitation and a mobile mass consistent with infective vegetation (Figure 3). A multidisciplinary approach was adopted, and the team confirmed the diagnosis of intracranial abscess attributed to IE. He was commenced on empiric intravenous vancomycin and gentamycin. Blood culture and cerebrospinal fluid culture drawn over 24 hours revealed methicillin-sensitive *S. aureus* growth. The culture sensitivity showed susceptibility to penicillin, and he was started on intravenous nafcillin and gentamycin. His condition improved gradually, and he became afebrile on hospital day four. He was well oriented in time, place, and person on hospital day seven. He was discharged with a one-and-a-half month course of intravenous antibiotics. On his recent follow-up, he was doing well.

## Discussion

IE is associated with frequent and several neurological complications, occurring in 20 to 40% of all patients with IE [1,2]. IE's neurologic complications can be divided into four main categories: hemorrhagic infarct, ischemic infarct, intracranial infection, and mycotic aneurysm. Among all the neurological complications, ischemic stroke is the most frequent complication accounting for 20-30% of all neurological complications. Brain abscess accounts for less than 1% of all neurological complications and is rarely reported in the literature.

Septic embolus leading to ischemic infarction is the leading cause of brain abscess. It may also arise due to direct extension of infection from the heart or contiguous arteritis. These septic emboli migrate to the brain's distal area and implant there, resulting in brain abscess and multiple ischemic infarctions. An intracranial abscess can be classified into a micro abscess and macro abscess based on the lesion's size, and micro abscess being more common is reported in cases of brain abscesses. Multiple microemboli with typical focal cerebral signs and symptoms have been reported in almost 11% of the clinical cases and more than 50% of the subject from a neuroradiologic case series [3,4]. The patient usually presents with non-localizing signs and symptoms, including decreased consciousness, encephalopathy, or psychosis. In micro abscess cases, patients develop signs and symptoms many days after ischemic stroke. The patients have a wide range of clinical presentations from asymptomatic to clinical deterioration signs [5].

IE leading to embolization in brain tissue is the prime cause of almost all neurological complications. Emboli to the brain are not so common and result in increased morbidity and mortality compared to other systemic emboli [1]. Cerebritis, meningitis, and abscess are the primary brain infections in patients with IE. Abscess formation is preceded by cerebritis, which is a demarcated area of parenchymal softening. However, pathologically, cerebritis involves scattered areas of necrosis, edema, vascular congestion, and perivascular inflammatory infiltrates. The progression of an area of cerebritis to an abscess formation involves liquefaction of the central zone of cerebritis and encircled by a collagen capsule [6,7].

**Figure 4:**
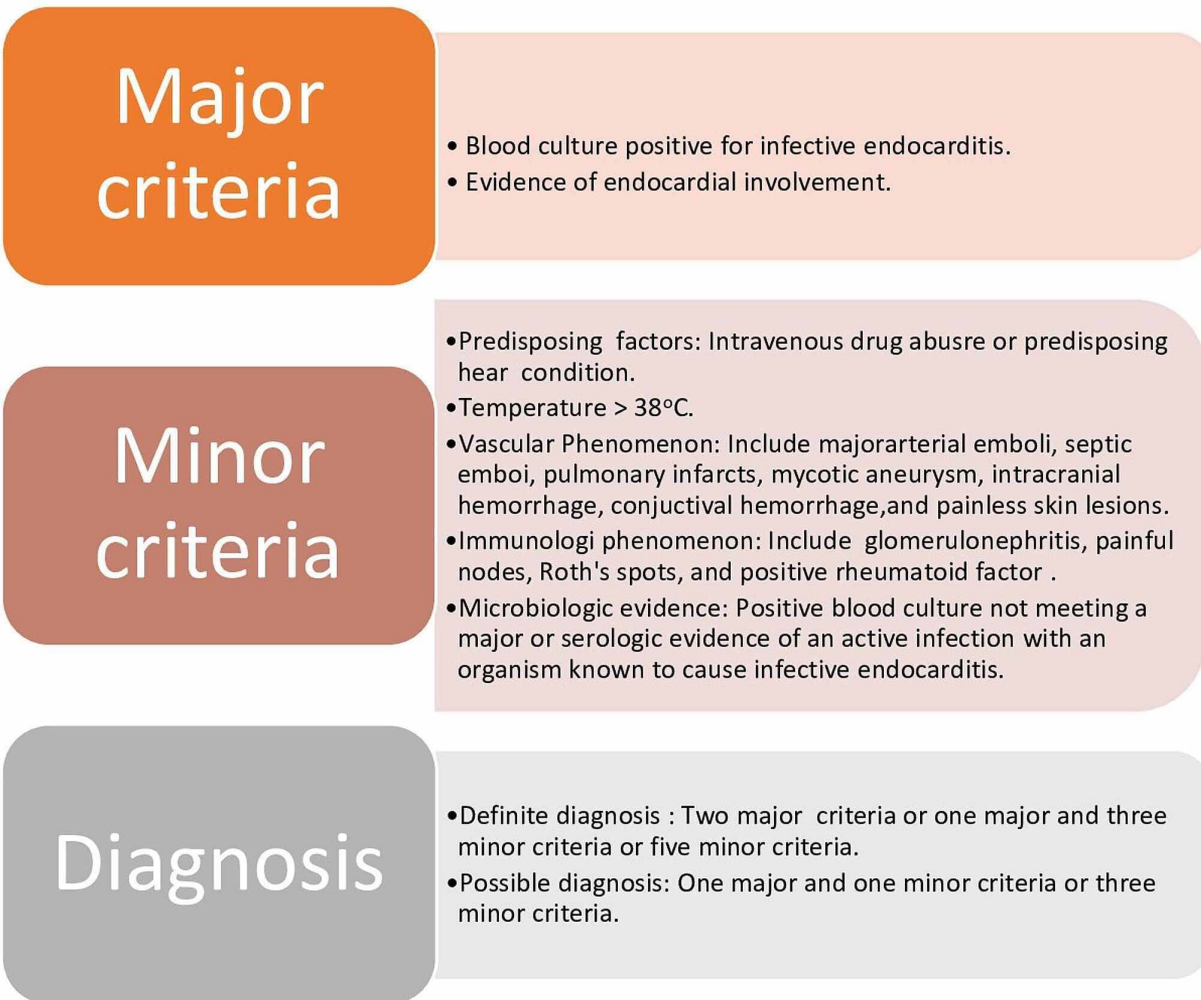
Duke criteria for infective endocarditis

The typical initial manifestations of IE include fever and fatigue. The patient can also present with nonspecific signs and symptoms such as myalgia, arthralgia, night sweats, and headache. The patients with multiple cerebral emboli have a relatively non-focal neurological examination, suggestive of meningitis and encephalitis. The patients with congenital heart disease often have persistent murmurs, and a new murmur on auscultation is less likely to support IE diagnosis in these patients. Diagnosis of IE is usually made by Duke Criteria (Figure 4). Over a 24-48 hour period, multiple positive blood cultures are compulsory for accurate diagnosis, and other common blood findings that aid in diagnosis is anemia and elevated ESR. Echocardiography helps in the diagnosis of IE by spotting vegetation.

MRI is more sensitive in IE-associated neurologic lesions [4]. MRI has four patterns of neurologic presentation in IE: (1) embolic infarctions, (2) multiple patch infarctions, (3) hemorrhagic infarctions, and (4) small nodular or ring enhancing white matter lesions [8].

Directed antibiotic therapy is the primary treatment of all neurologic complications of IE [9]. It has been reported that the risk of thromboembolism reduces rapidly after the first few days of suitable antibiotic treatment. The characteristics of vegetation and duration of antibiotics determine the risk of developing neurological complications. Thrombolysis is not recommended in patients with IE and acute embolic stroke due to asymptomatic emboli that have occurred more than three hours before starting the treatment. Secondary hemorrhages are the potential complications of patients with IE and cerebral abscess. Antiplatelet therapy has shown efficacy in reducing the risk of thromboembolism in IE and appears harmless in patients with IE and cerebral emboli [10].

## Conclusions

IE leading to intracranial lesions remains a complex scenario requiring a high index of suspicion, which warrants a multidisciplinary approach since the symptoms of an intracranial abscess may be non-focal. Rapid diagnosis and initiation of the appropriate antibiotics may be crucial for the management and secondary emboli prevention. IE is highly suggestive in patients with predisposing conditions and intracranial ring-enhancing lesions.
